# Optimization of 25% Sulfosalicylic Acid Protein-to-Creatinine Ratio for Screening of Low-Grade Proteinuria

**DOI:** 10.1155/2021/6688941

**Published:** 2021-01-28

**Authors:** Ambulugala Gamage Rajika Greshamali Jinadasa, Lanka Acharige Shalka Madushan Srimantha, Indika Deepani Siriwardhana, Kalani Buddika Gunawardana, Anoja Priyadarshani Attanayake

**Affiliations:** ^1^Department of Medical Laboratory Science, Faculty of Allied Health Sciences, University of Ruhuna, Galle, Sri Lanka; ^2^Department of Pathology, Faculty of Medicine, University of Ruhuna, Galle, Sri Lanka; ^3^Department of Biochemistry, Faculty of Medicine, University of Ruhuna, Galle, Sri Lanka

## Abstract

Proteinuria is an important prognostic marker in the diagnosis and management of kidney diseases. Sulfosalicylic acid method (SSA) is a simple, low cost, qualitative test, widely used to assess proteinuria. The aim of this study was to optimize SSA test as a quantitative screening tool to assess proteinuria at lower excretory levels which would facilitate the screening and early diagnosis of renal impairment using protein-to-creatinine ratio (PCR). The study was conducted in two phases. In phase I, optimum SSA percentage to detect low-grade proteinuria was selected by comparing the performance of 3%, 6%, and 25% SSA methods in manual spectrophotometric analysis. In phase II, clinical applicability of the optimized method was evaluated using retained urine samples of patients with chronic kidney disease (CKD) assessed for urine protein by the pyrogallol red (PGR) method in a tertiary care hospital in Sri Lanka. Optimized 25% SSA protein-to-creatinine ratio (PCR) was compared with PGR PCR and albumin-to-creatinine ratio (ACR). Sensitivity, specificity, degree of agreement, correlation, and diagnostic accuracy were evaluated. Turbidimetric analysis using 25% SSA was linear in the range 3–50 mg/dL giving the highest analytical sensitivity. The test yielded a sensitivity of 86.5% and specificity of 96.5% and a degree of agreement of 5 mg/dL with the PGR method. Optimal cut-off for 25% SSA PCR in receiver operating characteristic analysis was 166 mg/g. Spearman's correlation coefficient for 25% SSA PCR versus ACR was *r* = 0.823, *p* < 0.0001, and for 25% SSA PCR versus PGR PCR was *r* = 0.913, *p* < 0.0001. The 25% SSA PCR has a sensitivity of 92% against ACR, the current prognostic marker for proteinuria in patients with CKD. The 25% SSA test is a simple method, and it performs satisfactorily as a screening test with a cut-off for PCR optimized at 166 mg/g. The test merits further evaluation due to its low cost.

## 1. Introduction

Chronic kidney disease (CKD) has become a major healthcare problem all over the world with a global mean prevalence of 13.14% in 2016 [1]. With respect to this global prevalence, a remarkable increase of the disease has emerged in Sri Lanka within the past two decades [2]. Major etiologies for CKD are diabetes mellitus and hypertension. Meanwhile, a new form of the disease, with no identifiable etiology termed as chronic kidney disease of unknown etiology (CKDu) has emerged in several countries, including Sri Lanka [3]. International Society of Nephrology defines CKD as abnormalities of kidney structure or function persisting for more than three months with implications for health leading to dialysis and kidney transplant in the final stage [4]. Diagnosis of renal damage in the early course of the disease would prevent its development to the chronic irreversible stage and facilitate better prognosis. Thus, sensitivity in screening and specificity in diagnostic methods of CKD play a major role in disease management. Among the available screening techniques, persistent proteinuria has become a key evaluation target in assessing kidney function and progressive kidney damage. It refers to urinary protein excretion ≥150 mg/24 hours. Albuminuria (albumin-to-creatinine ratio (ACR) ≥3 mg/mmol or 30 mg/g) is a common finding in CKD due to diabetic nephropathy. Assessment of protein concentration in 24-hour urine collection is considered as the gold standard for proteinuria estimation [5]. However, sample collection for 24-hour test has limitations. Kidney Disease Improving Global Outcomes (KDIGO) guidelines states estimated glomerular filtration rate (eGFR) and ACR as the recommended evaluation criteria for the classification of CKD [4]. Furthermore, it is mentioned that eGFR begins to decline following a greater damage (nearly 50% damage) to renal tissues [6]. Thus, ACR is a better marker over eGFR for early diagnosis of renal damage. Dipsticks are currently being used in population screening programmes for CKD, but dipsticks are more sensitive to urine albumin. Dye binding methods, like pyrogallol red (PGR) and coomassie brilliant blue, and immunoturbidimetric methods are being used at clinical laboratories for quantitative analysis of urine protein. Cystatin–C, kidney injury molecule-1 (KIM-1), neutrophil gelatinase-associated lipocalin (NGAL), and alpha 1 microglobulin (A1M) are more specific urine biomarkers which are being evaluated in research [7]. However, the requirement of expensive chemicals and analyzers for some of these have limited their use in hospital laboratory set up and these are not cost-effective for community based screening [2, 8]. Thus, cost-effective, convenient screening methods could immensely contribute to disease management and early diagnosis of CKD and CKDu patients.

Sulfosalicylic acid (SSA) method is a noninvasive, cost-effective turbidimetric test routinely used for semiquantitative evaluation of proteinuria up to 500 mg/dL in clinical laboratories. Addition of SSA to an acidified protein solution results in denaturation of proteins followed by precipitation. The content of protein can be estimated semiquantitatively using a grading scale or quantitatively by measuring the turbidity. It has long been used for quantitative evaluation of total protein in urine and cerebrospinal fluid before dye-binding methods, such as PGR, were introduced [9].

This study was aimed to investigate the feasibility of using SSA turbidimetric method for quantitative evaluation of proteinuria in the lower range and to assess its clinical applicability by assessing protein-to-creatinine ratio (PCR) which minimizes the effects of factors, like pyrexia, status of hydration, and strenuous exercises. We were interested to optimize it for the lower range (0–50 mg/dL) since it would facilitate the detection and the follow-up of the progression of disease at early stages using a low-cost screening method. It is important to evaluate protein-to-creatinine ratio (PCR) and compare against standard ACR since PCR and ACR provide better clinical information of kidney disease rather than assessing total urine protein concentration in a random urine sample [10–12]. The current gold standard for assessing urine protein is PGR dye binding method, which requires expensive reagents and automated analyzers, which constrain its usability as a screening test. Therefore, development of SSA test to assess PCR as a reliable quantitative low-cost screening method could be useful in early diagnosis of renal impairment.

## 2. Materials and Methods

SSA test method is used in the clinical setup with various modifications. Different concentrations of SSA (3%, 6%, and 25%) have been used in the current laboratory setup for semiquantitative estimation of urine protein.

Therefore, in this study, we had to address two basic research questions. First, can we use SSA for quantitative estimation of urine protein in the lower range, and if so, what is the optimum percentage? Second, is it clinically applicable? In order to address these two questions, we conducted the study in two phases using convenient sample method. In phase I, SSA method was optimized to quantitate urine protein in the lower range (0–50 mg/dL), and the clinical validity of the optimized method was assessed using samples received from the Nephrology Clinic at Teaching Hospital, Karapitiya, Sri Lanka, for analysis of urine protein by PGR method. In phase II, the clinical applicability of the optimized method was evaluated by comparing urine PCR with gold standard urine ACR on samples from CKD patients [4]. Ethical approval for the study was granted by the Ethics Review Committee of Faculty of Allied Health Sciences, University of Ruhuna, Galle, Sri Lanka (14.02.2018.005).

Retained urine samples of patients aged 18–65 years, admitted to wards and/or attending the Nephrology Clinic at Teaching Hospital, Karapitiya, Sri Lanka, were used for this study. They were already analyzed by PGR dye binding method using Thermo Scientific Indiko Plus Fully Automated Biochemistry Analyzer (Finland) for urine protein concentration. Samples with urine total protein ≤20 mg/dL and ACR ≤30 mg/g were considered as normal, in phase I and II, respectively. In identification of CKD patients, serum creatinine value measured within the past three months was collected from laboratory information system, and eGFR was calculated using CKD/EPI formula according to 2012 KDIGO guidelines [4].

### 2.1. Phase I Method Development

This phase was conducted to find out the most suitable concentration of SSA to quantitatively evaluate urine protein in the lower range. Therefore, standard curves were prepared using 3%, 6%, and 25% SSA in a dilution series of bovine serum albumin factor V using manual spectrophotometric analysis. Absorbance was used to generate standard curves. The optimum SSA percentage was selected depending on analytical sensitivity (gradient of the standard curve), lowest detection limit, and linearity range.

### 2.2. Preparation of Standard Curves

A dependent dilution series of albumin standard (2.125–200 mg/dL) was prepared. This was used for the generation of albumin standard curve [13]. Albumin standard of 2.0 mL was acidified with 50 *μ*L of trichloroacetic acid followed by 100 *μ*L of SSA at 25 ± 2°C. Proteins were allowed to precipitate for three minutes, and the absorbance was measured at 600 nm against reagent blank in a double beam spectrophotometer (SHIMADZU 1800 UV-VIS, Japan) using manual procedures. Standard curves were generated for 3%, 6%, and 25% SSA assays by plotting absorbance verses concentration of albumin standards.

### 2.3. Clinical Validity of Standard Curve

**Figure 1:**
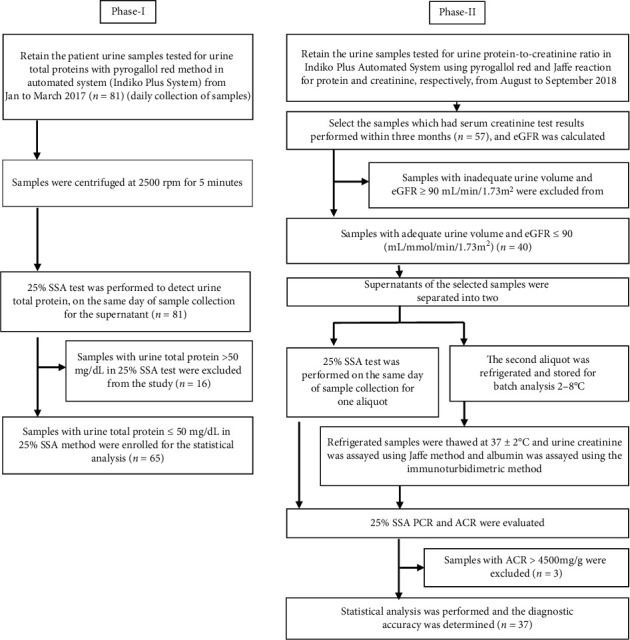
Flow diagram of the study.

A comparative cross-sectional study was performed to assess the performance of the selected SSA percentage on clinical samples. Protein concentrations of 81 urine samples were tested on the same day of sample collection with modified SSA method. Two (2) mL of urine sample was initially acidified with 50 *μ*L of trichloroacetic acid followed by addition of 100 *μ*L of SSA, and the turbidity was measured as described above. Sensitivity, specificity, positive predictive value (PPV), and negative predictive value (NPV) were evaluated considering PGR method as the gold standard. Since patient samples were used for the study, samples with proteinuria less than 20 mg/dL measured by the PGR method were considered as normal population for the study. Intra-assay precision was assessed using albumin spiked normal urine sample at the lower range (20 mg/dL) and a pooled patient sample at the upper range (40.6 mg/dL). Bland-Altman plot and linear regression were used to assess the degree of agreement and correlation between the two methods, respectively (Figure 1).

### 2.4. Phase II Assessment of Clinical Applicability

Phase II was designed to address the second research question: to check the clinical applicability of the optimized SSA test. In this phase, PCR was evaluated using modified SSA method and PGR method, and they were compared with ACR in a receiver operating characteristic (ROC) curve. Serum creatinine test results were available in 57 patients. However, 17 urine samples were excluded due to the inadequacy of urine sample volume and eGFR ≥90 mL/min/1.73 m^2^. A comparative cross-sectional study was performed using a sample of 40 CKD patients with persistently low eGFR (eGFR <90 mL/min/1.73 m^2^ for the past three months was considered as CKD) (Table 1. Background data of the patients are attached in Supplementary Material (available here).

PCR and ACR were evaluated and compared for CKD patients. The samples were tested for urine total protein with modified SSA turbidimetric method, creatinine with Jaffe reaction using manual spectrophotometric analysis, and albumin with immunoturbidimetric method in BTS 350 semiautomatic biochemistry analyzer (Spain). Urine creatinine and albumin were measured in a batch analysis of refrigerated samples thawed at 37 ± 2°C. Assessors were not blind to clinical data, reference, and index test results. Albumin standard was traceable to EMR-DA 470. ACR and SSA PCR were measured. Cut-off for PCR was 150 mg/g and ACR was 30 mg/g. Samples with ACR ≤30 mg/g were considered as normal [4]. Inter- and intra-assay precisions and percentage inaccuracies were calculated in method validation. Albumin spiked normal urine samples of two levels (10 mg/dL and 30 mg/dL) were used for precision checks in SSA method, and manufacturer-provided standards were used to evaluate percentage inaccuracies. Performance characteristics, correlation studies, and diagnostic accuracy in ROC curve were assessed in statistical analysis [14]. Urine protein evaluation was performed on fresh urine samples as in phase I, and creatinine was measured by kinetic Jaffe method. IBM SPSS 20 statistical software was used for data analysis.

## 3. Results

### 3.1. Phase I Method Development

The best standard curve to detect protein concentration in the lower range was selected by assessing the analytical sensitivity (gradient of the standard curve) and the linearity range and the lowest detection limit of each standard curve generated for SSA percentages of 3%, 6%, and 25%, respectively (Table 2).

**Figure 2:**
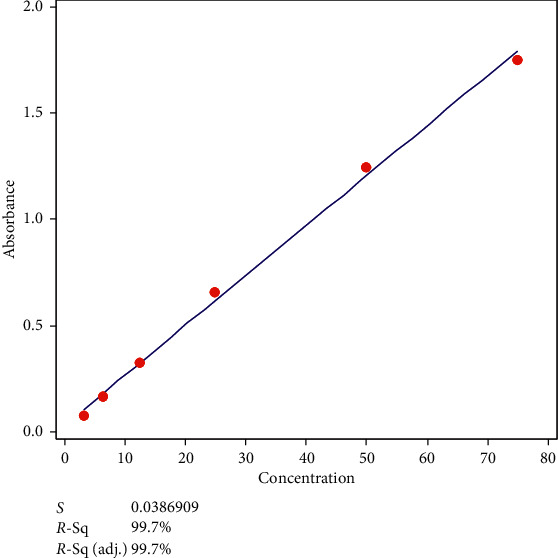
Standard curve of absorbance versus concentration of standard (bovine albumin) with 25% sulfosalicylic acid method. *S*: standard error of the estimate; *R*-Sq.: *R*-squared value; *R*-Sq. (adj.): adjacent *R*-squared value.

Out of the three standard curves, 25% SSA showed the highest sensitivity, having the highest gradient for detection of protein in the lower range (Figure 2). The linearity range was 3–50 mg/dL. An intra-assay precision of 7.3% and 4.5% was observed for urine protein concentrations at 20.0 mg/dL and 40.6 mg/dL, respectively.

To validate the optimized 25% SSA curve, a clinical study was performed using 81 patient samples (Table 3). The assessment of clinical validity at 95% confidence interval (CI) showed a sensitivity of 86.5% (95% CI 74.4–93.63) and specificity of 96.5% (95% CI 81.3–99.9), PPV of 97.8% (95% CI 87.6–99.9), NPV of 80.0% (95% CI 63.8–90.2), and accuracy of 90.1% (95% CI 81.4–95.1) when compared with the PGR method at a cut-off of 20 mg/dL for proteinuria.

The patient data deviated from normal distribution with skewness 0.968 and kurtosis 0.200; outliers were removed and statistical analysis was performed. In linear regression, a positive correlation (*r*) of 0.9023 (*R*^2^ = 0.8143) was observed between 25% SSA method and PGR method. The equation of [PGR] = 1.4395 [SSA] was derived through linear regression.

**Figure 3:**
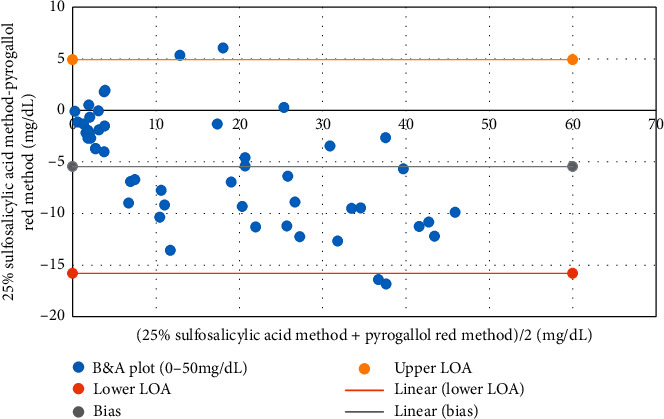
Bland–Altman plot of pyrogallol red method versus 25% sulfosalicylic acid method. B&A plot: Bland-Altman plot; lower LOA: lower limit of agreement; upper LOA: upper limit of agreement.

The degree of agreement between the SSA method and PGR method was evaluated in Bland-Altman analysis. Data range used was 3–50 mg/dL. The plot showed a bias of −5.5 mg/dL and a SD of 5.3 mg/dL. The lower limit and the upper limit of agreement were −15.8 and 4.9 mg/dL, respectively (Figure 3).

### 3.2. Phase II Study of Clinical Applicability

Phase II was conducted to check the clinical applicability of 25% SSA method. Urine PCR of CKD patients were evaluated using 25% SSA test and compared with PGR PCR and ACR, which is the recommended test for diagnosis and classification of CKD. Inter-assay and intra-assay precisions of albumin spiked in-house quality control samples were assessed at 10 mg/dL and 30 mg/dL levels of albumin concentration (Table 4).

The performance characteristics of 25% SSA PCR were derived at a cut-off of 150 mg/g for proteinuria considering ACR as the gold standard at cut-off 30 mg/g. The evaluated performance characteristics observed were sensitivity 92.6% (95% CI 75.5–99.0), specificity 69.2% (95% CI 42.0–87.6), PPV 86.2% (95% CI 68.8–95.2), NPV 81.8% (95% CI 51.1–96.0), and accuracy 85% (95% CI 70.5–93.3) against ACR (Table 5).

The sample data were nonparametrically distributed (skewness 3.332; kurtosis 11.54). Therefore, Spearman's correlation statistic was performed. Spearman's correlation between ACR and 25% SSA PCR is r = 0.823, *p* value <0.0001. Spearman's correlation between ACR and PGR PCR, is r = 0.864, *p* value <0.0001. Also, Spearman's correlation between 25% SSA PCR and PGR PCR is r = 0.913, *p* value <0.0001. Therefore, 25% SSA PCR shows a significant positive correlation with ACR and PGR PCR at 0.01 level (2-tailed).

**Figure 4:**
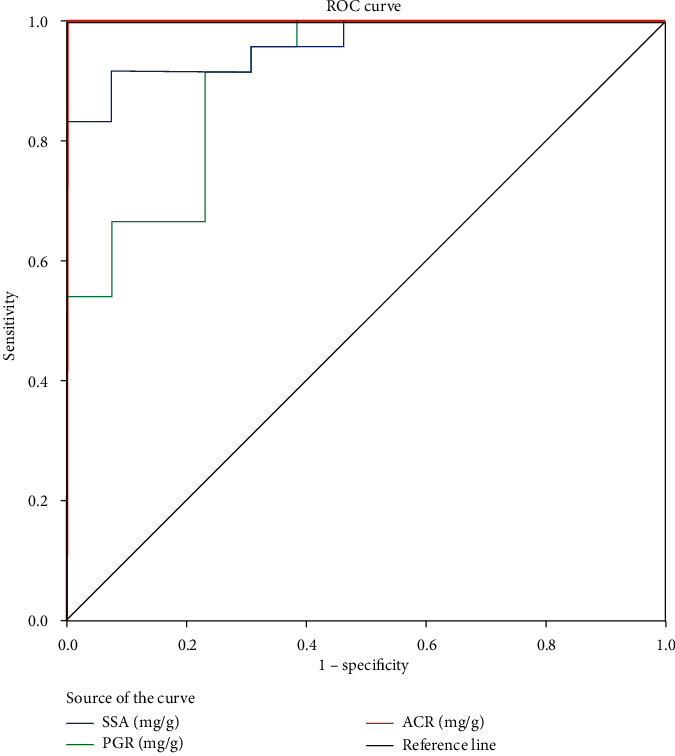
The summary plot of receiver operating characteristic curve in which 25% sulfosalicylic acid protein-to-creatinine ratios were compared with pyrogallol red protein-to-creatinine ratios in clinical applicability test. SSA: sulfosalicylic acid; PGR: pyrogallol red; ROC: receiver operating characteristic curve.

Three outliers were removed, and 37 patient data were used in the statistical analysis. A receiver operative characteristic curve was evaluated to determine the diagnostic accuracy, using area under the curve (AUC) of the 25% SSA PCR relative to ACR as the gold standard (Figure 4). Optimal cut-off was derived with best sensitivity and specificity using patient data.

AUC observed for SSA and PGR was 0.904 (95% CI, 0. 803–1.00) and 0.962 (95% CI, 0.908–1.00), respectively. AUC being above 0.5 indicates that both SSA and PGR methods are effective in identifying the presence of a disease. The optimal cut-off derived for 25% SSA method from ROC analysis is 166 mg/g at 95% CI of sensitivity and specificity. This is closer to the currently accepted cut-off for PCR of 150 mg/g in detecting proteinuria [4].

## 4. Discussion

In the this study, conventional qualitative 25% SSA test for urine protein measurement was optimized as a low cost clinically applicable sensitive test by evaluating protein-to-creatinine ratio for the quantitative determination of urine proteins at lower excretory levels. The cost evaluation studies show that cost per test of 25% SSA method is 0.27 LKR, whereas, for PGR method and for microalbumin method, it is 25.00 LKR and 168.00 LKR, respectively, in Sri Lankan rupees. An optimal cut-off of 166 mg/g was derived for PCR in 25% SSA method which is closely associated with the current cut-off for PCR of 150 mg/g in diagnosing proteinuria [10–12].

Urine protein and creatinine were assayed using the 25% SSA and Jaffe methods, respectively, using spectrophotometric analysis. Albumin was measured in immunoturbidimetric method in BTS 350 semiautomatic system. The 25% SSA test was performed on the same day of sample collection. Manually measured creatinine values were used to calculate 25% SSA PCR and ACR. The PGR PCR were derived from Indiko Plus Fully Automated Biochemistry Analyzer at the Pathology Laboratory, Teaching Hospital, Karapitiya, Sri Lanka.

A previous study has derived an optimum cut-off of 160 mg/g for PCR, which is closer to 166 mg/g when SSA was used to assess urine protein and Jaffe method for creatinine [11]. Therefore, the current study has been able to regenerate similar figures in a different laboratory setting. Limited sample numbers were used in the project depending on the feasibility within the limited time frame. However, similar sample numbers have been used in previous method comparison studies for urine protein measurement [8, 15]. KDIGO guidelines recommend eGFR together with ACR for diagnosis and classification of CKD [4]. Nevertheless, ACR targets only a fraction of total protein amount excreted in urine. The 25% SSA test could be clinically applicable as a screening test in early diagnosis of proteinuria (both albumin and nonalbumin) because of its improved sensitivity at lower levels of protein excretion. Tubulointerstitial diseases of kidney predominantly give rise to nonalbumin proteinuria, which is not detected by ACR. CKDu is an example where tubulointerstitial damage predominates [16].

CKDu has shown an alarming rise in prevalence among the rural agricultural communities in Sri Lanka over the last three decades [17]. The disease has been reported among similar socioeconomic communities around the globe in the developing world [3]. Early diagnosis of the disease is important to ensure better patient outcomes. Therefore, a sensitive and cost-effective test for early identification of tubular proteinuria is invaluable in the early diagnosis of CKDu. Although newer biomarker assays, molecular markers, and histopathological tests have been found to be more sensitive and specific for accurate diagnosis of CKDu, sophisticated technology, expensive reagents, competent human resources, and invasive sampling methods have limited their use in population screening [7, 18–21]. A large-population-based study conducted in Sri Lanka in the year 2015 has recommended the use of combined eGFR and urine ACR or urine PCR (UPCR) as screening tests for early identification of CKDu [22]. The performance of eGFR and urine based screening tests have been studied among the population at risk. Dipstick proteinuria, urine ACR, UPCR using PGR method, and semiquantitative screening test for urine protein detection using SSA had been used in the aforementioned study. The aforementioned large-population-based study has revealed that the sensitivity of UPCR and semiquantitative SSA test were superior to that of ACR and dipstick protein test, in screening for CKDu [22]. Thus, combined evaluation of PCR using quantitative SSA test, as observed in our study (25% SSA PCR), would be effective in screening programmes for CKDu.

The modified 25% SSA PCR test is a simple and cost-effective test for identification of low-grade proteinuria which appears early in tubulointerstitial diseases. The test is easily applicable in population screening programmes for CKD/CKDu to identity low-grade proteinuria. However, measurement of urine protein concentration alone is not clinically significant. Therefore, evaluation of PCR in a random sample is essential in clinical diagnosis. In addition, PCR is highly correlated with 24-hour protein excretion, which is the recommended sample of urine protein estimation [10–12]. The 25% SSA test in our study has been validated and its clinical applicability in PCR has been assessed.

Limitations of this method include the requirement of fresh urine samples; the test is nonresponsive in refrigerated urine samples. The current study was conducted in manual spectrophotometric analysis. Applicability of the test on semiautomated or automated systems with minimum sample volume should be evaluated for it to be applicable for population screening. The test needs further evaluation as a screening test among CKDu endemic and nonendemic regions to further determine its clinical applicability.

## 5. Conclusion

In conclusion, the 25% SSA PCR method has a sensitivity of 92% against ACR, the current prognostic marker for proteinuria in patients with CKD. The test, with its ability to identify patients with low-grade proteinuria optimized at a cut-off 166 mg/g, merits further evaluation as a screening test due to its cost-effectiveness and high sensitivity.

## Figures and Tables

**Table 1:** eGFR distribution within the sample population in phase II.

eGFR category (mL/mmol/min/1.73 m^2^)	Number of patients
60–90	13
44–59	11
30–45	12
15–29	3
<15	1

eGFR: estimated glomerular filtration rate calculated depending on CKD/EPI creatinine equation.

**Table 2:** Analytical sensitivity (gradient) and linearity range of 3%, 6%, and 25% sulfosalicylic acid for albumin standard curves, where the protein concentrations were assessed in the modified SSA quantitative method.

SSA percentage (%)	Gradient (slope/sensitivity)	Linearity range (mg/dL)
3	0.0109	4–40
6	0.0196	4–40
25	0.0241	3–50

SSA: sulfosalicylic acid.

**Table 3:** Patient data for the calculation of performance characteristics of the 25% sulfosalicylic acid protein concentration versus pyrogallol red protein concentration.

	Diseased (PGR protein value ≥20 mg/dL)	Nondiseased (PGR protein value <20 mg/dL)
25% SSA (protein value ≥20 mg/dL)	45	1
25% SSA (protein value <20 mg/dL)	7	28

SSA: sulfosalicylic acid. PGR: pyrogallol red.

**Table 4:** Precision check data of 25% sulfosalicylic acid method in phase II.

	No. of samples	Intra-assay			Interassay	
		Mean (mg/dL)	SD	CV%	Mean (mg/dL)	SD
10 mg/dL	20	9.82	0.45	4	9.65	0.46
30 mg/dL	20	30.06	0.99	3	29.61	0.81

SD: standard deviation; CV: coefficient of variation.

**Table 5:** Patient data for the calculation of performance characteristics of the 25% sulfosalicylic acid protein-to-creatinine ratio (SSA PCR) versus albumin-to-creatinine ratio (ACR).

	Diseased (ACR ≥30 mg/g)	Nondiseased (ACR <30 mg/g)
25% SSA PCR ≥150 mg/g	25	4
25% SSA PCR <150 mg/g	2	9

25% SSA PCR: 25% sulfosalicylic acid protein-to-creatinine ratio; ACR: albumin-to-creatinine ratio.

## Data Availability

The data used to support the findings of the present study are available from the corresponding author upon request.

## Supplementary Materials

Supplementary Materials The authors have used STARD guidelines for reporting the research work since this study involves the optimization of a diagnostic test. Therefore, the STARD checklist is uploaded as a supplementary material. A table with background data of the patient sample used in phase II is uploaded for further clarification.
